# Percepção dos profissionais das unidades de pacientes críticos sobre morte encefálica

**DOI:** 10.5935/0103-507X.20210063

**Published:** 2021

**Authors:** Neide da Silva Knihs, Sibele Maria Schuantes Paim

**Affiliations:** 1 Departamento de Enfermagem, Universidade Federal de Santa Catarina - Florianópolis (SC), Brasil.; 2 Escola Paulista de Enfermagem, Universidade Federal de São Paulo - São Paulo (SP), Brasil.

**To the Editor**

The health teams in critical patient units (intensive care units, emergency services and units with critically ill patients) experience the initial process of organ donation in their hospital routines. In these units, patients with clinical signs of brain death (BD) are diagnosed and approved or not for donation, depending on the wishes of the family.^(1^-^4)^

Given this reality, challenging situations arise because the professionals on these teams are faced with patients who only have vital signs because they are artificially maintained alive. This profoundly disrupts the social, cultural and professional values in the face of death, leading the team to confront, on the one hand, the confirmed death of a human being and a family impacted by the death of the family member and, on the other hand, a potential donor who can save patients on donor waiting lists.^(3, 5^-^8)^

This study aimed to identify the perception of professionals in critical patient units about the diagnosis of BD, organ donation, and the delivery of the body to the family in case the family refuses to donate organs.

This was a quantitative and cross-sectional study conducted in six hospitals that are reference centers for organ donation in southern Brazil.

The study participants were professionals who worked in critical patient units. The inclusion criteria were as follows: professionals who worked in patient care, implemented the BD protocol and treated potential organ and tissue donors for more than 3 months. Professionals on vacation, maternity leave, sick pay, and sick leave during the data collection period were excluded, as were those from other units who were only covering for those on vacation. The sample was calculated considering a significance level of 95%, and it consisted of 653 professionals.

Before collecting information, the researchers contacted the coordinators of the units. After agreeing to participate in the study, the participants signed an informed consent form.

Data collection occurred between November 2017 and October 2018 and was performed by researchers and undergraduate students using a questionnaire with seven questions, four of which were related to professional profile and three of which addressed the belief in the diagnosis of BD, the option for donation, and decision-making. For each question, there were only two answer options: yes or no.

The data were analyzed using the absolute (n) and relative (%) frequency distributions. The quantitative variables are presented as the mean, 95% confidence interval, median, minimum, maximum, and standard deviation.

This study was approved by the Research Ethics Committee (CAAE 54562616.1.1001.0121).

A total of 653 professionals from critical patient units answered the questionnaire, including 276 (42.3%) nurses, 217 (33.2%) nursing technicians, 130 (19.9%) physicians, and 30 (4.6%) other professionals on multidisciplinary teams (psychologists and physiotherapists).

The answers to the questions are shown in table 1. Notably, 15 (2.3%) professionals did not believe in the diagnosis of BD, 136 (20.8%) were not organ or tissue donors, and 251 (38.4%) would not turn off the equipment and hand over the body to the family in case of family refusal to donate.

In recent years, several studies have been conducted addressing the stages of the process of organ and tissue donation; however, they focus only on the family refusal to donate.^(9, 10)^ Few studies have investigated the reality of health professionals considering the stages of the process of organ and tissue donation.^(11, 12)^

These professionals are ordinary individuals, with their own values, principles, and beliefs related to the topic of death and organ donation. They are people from different social and cultural backgrounds, and each one of them has his or her own opinion on this topic. The relationship of teams in critical patient units with this subject, especially doctors and nursing staff, goes beyond current standards and legislation and enters into the complexity of social beings, with professionals immersed in feelings, emotions and decisions.^(5)^

From the perspective of the experience of BD, there are professionals who consider this death to be a crime committed by society.^(6)^ Even after extensive discussion of this topic across the years, BD is seen as a wake at bedside, and there are distinct paradoxes between professionals and family members regarding the meaning of death when it is confirmed through the diagnosis of BD. Such implications may influence the care provided to the potential donor and family members by professionals in critical patient units.^(5^.^13)^

All these factors experienced in this process may be associated with the results of this study. Professionals have many doubts and questions, in addition to the fear and uncertainty of facing the family when faced with any decisions they have to make, especially when it involves disconnecting equipment and returning the body to the family in case it is impossible to donate organs and tissues. The decision of these participants may be based on the experiences of this process in critical patient units, in the disbelief in the process of organ donation, in the different meanings of BD, in the little knowledge of the current legislation and in moral, ethical, religious and cultural values.^(3, 5, 13)^

**Table 1:** Responses by members of critical care unit teams regarding the diagnosis of brain death, choice of being an organ donor and delivery of the body to the family in case of family refusal to donate

Question	Nurse n (%)	Nursing technician n (%)	Physician n (%)	Other n (%)
Do you believe in the diagnosis of BD?				
Yes	270 (97.83)	207 (95.39)	129 (99.23)	30 (100)
No	05 (1.81)	09 (4.15)	01 (0.77)	00 (00)
No response	01 (0.36)	01 (0.46)	00 (00)	00 (00)
Total	276 (100.0)	217 (100.0)	130 (100.0)	30 (100.0)
Are you an organ donor?				
Yes	239 (86.60)	140 (64.52)	110 (84.62)	23 (76.67)
No	36 (13.04)	75 (34.56)	19 (14.61)	06 (20.00)
No response	01 (0.36)	02 (0.92)	01 (0.77)	01 (3.33)
Total	276 (100.0)	217 (100.0)	130 (100.0)	30 (100.0)
Would you disconnect the equipment and return the body to the family?				
Yes	166 (60.14)	100 (46.08)	94 (72.31)	14 (46.67)
No	103 (37.32)	98 (45.16)	35 (26.92)	15 (50.00)
No response	07 (2.54)	19 (8.75)	01 (0.77)	01 (3.33)
Total	276 (100.0)	217 (100.0)	130 (100.0)	30 (100.0)

BD - brain death.

The aim of this study was to identify whether the professionals who worked in critical patient units believed in the diagnosis of BD, were organ and tissue donors, and would deliver the body to the family if it was not possible to donate organs and tissues. Most professionals showed clarity in the aspects investigated, but there are still a large number of these professionals who are not donors and, when asked, say that they do not turn off equipment and deliver the body to the family when donation is not possible.

Thus, the results from this study show that among the professionals who conduct steps in the donation process, there are people who are not familiar with the process and make different decisions. These results indicate that administrative levels should investigate such realities to propose internal actions that enable more comfort, space, and support to professionals as well as to promote more training on the subject. It is also necessary to increase the production of scientific knowledge in this area as a way to support other agencies and managers, avoiding emotional and psychological overload, in addition to dilemmas and ethical conflicts within professionals, colleagues and the family.
